# The Study of SLC26A4 Gene Causing Autosomal Recessive Hearing Loss by Linkage Analysis in a Cohort of Iranian Populations

**Published:** 2014

**Authors:** Somayeh Reiisi, Mohammad Hosein Sanati, Mohammad Amin Tabatabaiefar, Shahla Ahmadian, Salimeh Reiisi, Shahrbanoo Parchami, Hamid Porjafari, Heshmat Shahi, Afsaneh Shavarzi, Morteza Hashemzade Chaleshtori

**Affiliations:** 1 Medical Genetics Department, National Institute of Genetic Engineering and Biotechnology (NIGEB).; 2 Medical Genetics Department, Ahvaz Jundishapur University of Medical Sciences, Ahvaz, Iran.; 3 Cellular and Molecular Research Center, Shahrekord University of Medical Sciences, Shahrekord, Iran.; 4 Biochemistry Department, Maleke-Ashtar University of Technology, Tehran Iran.; 5 Medical Genetics Department, Hamedan University of Medical Sciences, Hamedan, Iran.

**Keywords:** SLC26A4, hearing loss, linkage analysis, Iran

## Abstract

Sensorineural non-syndromic hearing loss is the most common disorder which affects 1 in 500 newborns. Hearing loss is an extremely heterogeneous defect with more than 100 loci identified to date. According to the studies, mutations in *GJB2* are estimated to be involved in 50- 80% of autosomal recessive non-syndromic hearing loss cases, but contribution of other loci in this disorder is yet ambiguous. With regard to studies, DFNB4 locus (*SLC26A*4) can be classified as the second cause of hearing loss. So, this study aimed to determine the contribution of this locus in hearing loss as well as the frequency of SLC26A4 gene mutations in a population in the west of Iran. In this descriptive laboratory study, we included 30 families from the west of Iran with no mutation in GJB2 gene. Linkage analysis was performed by DFNB4 (SLC26A4) molecular markers (STR). The families with hearing loss linked to this locus were further analyzed for mutation detection. SLC26A4 gene exons were amplified and analyzed using direct DNA sequencing. In studied families, 2 families displayed linkage to DFNB4 locus. Identified mutations include mutation in exon 5 (c.416 G>T) and in splicing site of exon 7 (IVS-2 A>G or c.919-2 A>G).

Autosomal recessive non- syndromic hearing loss (ARNSHL) is the most common defect at birth, which affects 1 in 500 newborns (1). Deafness is a heterogeneous disorder and can occur due to genetic factors or environmental factors or both (2). Over 50% of hearing loss (HL) causes are attributed to genetic factors of which 70% are non-syndromic (3). Up to now, more than 60 genes have been identified for non- syndromic hearing loss (4). Since HL is highly heterogeneous, researchers suggest studying large size families with different ethnicities such as the Middle East populations (5). Based on these studies, DFNB1 locus is the most common cause of HL and accounts for up to 50% of cases in Asia, Europe and northern America (6). Studies in Iran also have indicated that the contribution of *GJB2* gene in HL in different ethnicities in Iran is different ranging from 27-38% in a population of north of Iran to 0-4% in the south east of Iran (7, 8). Up to now, the relative contribution of mutations in other genes associated with HL has been identified only in the limited number of Iranian families. To determine the exact contribution of each of these genes in Iranian families, more comprehensive studies are required (-). For recessive deafness, the most frequ-ently involved genes include GJB2, SLC26A4, MYO15A, OTOF, CDH23 and TMC1 worldwide.

*SLC26A4* gene at DFNB4 locus is the second leading cause of HL. This gene consists of 21 exons and is located at 7q22-q31 (DFNB4), encoding Pendrin. Pendrin is an iodide/ chloride transporter that is expressed in cochlea, thyroid and kidney. This protein transports anions such as HCO, OH^-^, I^-^. Mutation in this gene is the second cause of HL which can cause ARNHL and Pendred Syndrome (PS). Signs and symptoms in PS are profound sensorineural HL along with enlarged thyroid or small sized thyroid. DFNB4 and PS have been seen in 1-8% of prelingual hearing loss cases (9). Mutations in this gene have been reported in different American, European and Asian populations such as Iran (12, 13). So far, more than 200 mutations in *SLC26A4* gene have been identified which are scattered in the whole gene and most of them are missense (http://www.healthcare-uiowa.edu/labs/pendredandbor/slcMutations.htm). These mutations are the most common causes of ARNHL in Iran and worldwide after *GJB2 *(14). This study aimed to determine the frequency of mutations and perform an analysis of the locus, due to the importance of DNBF4 after DNBF1 locus and also the frequency of mutations in studied cohort.

## Material and Methods

In this descriptive laboratory study, informational questionnaires were filled out and clinical evaluations were performed. We included 30 families with at least 2 ARNHL individuals from the western provinces of Iran with no Connexin 26 mutation detected in a previous research (7). After obtaining written consent from patients and their families, 5 ml peripheral blood from all members of families in tubes containing 0.5M EDTA. Genomic DNA was extracted from blood samples using standard phenol-chloroform protocol. The quantity and quality of extracted DNA were evaluated by using a spectrophotometer (UNICO 2100, USA) (15). For linkage analysis of the locus, 4 different STR markers were used. Upon encountering an uninformative marker, further markers were genotyped. The used markers and their charact-eristics are shown in table 1. The criteria for selecting these markers include greater heterozygosity values, shorter amplicon and locating near the known locus. Choice of STRs and their primers were based on their physical distance in NCBI Map Viewer and NCBI UniSTS. Polymerase chain reaction (PCR) amplification of markers, touchdown program was carried out with conditions described previously (16). For S-Link and LOD score calculation, the Easylinkage plus genetic software version 5.05 was used (17). To calculate S-Link, FastSlink version 2.51 was used. Two point and multi-point parametric LOD scores were calculated by Superlink version 1.6 and Genehunter version 2.91. For LOD score calculations, inheritance pattern of autosomal recessive, complete penetrance and allele frequency of 0.001 were assumed. Haplopainter software version 029.5 was used for reconstruction of haplotypes (18). For mutation analysis, 21 exons and intron-exon boundaries of *SLC26A4* gene were amplified using specific designed primers (19). Reaction conditions for amplification of exons in a volume of 50 μL were as follows: 5 μL 10x PCR buffer, 2 μL of 50 mM MgCl2, 1 μL of 10 mM dNTPs mix, 0.5 μL of each 50 pM forward and reverse primers, 2 μL of genomic DNA )100ng (and finally 0.5 U of Taq DNA polymerase enzyme. PCR was performed with following conditions: an initial denaturation at 95 ºC for 5 min followed by next 35 cycles of 94 ºC for 1 min (denaturation), annealing at 58-62 ºC (depending on exon) for 1 min, 72 ºC for 1 min (extension) and final extension at 72 ºC for 5 min. To detect any variant in the gene sequence, direct sequencing of amplified exons was performed bidirectionally using ABI 3730XL sequencing instrument.

**Table 1:** STR markers used and their characteristics

STR	Forward primer (5' 3')	Reverse primer (5' 3')	Size (bp)	Heterozygosiy
D7S2420	CCTGTATGGAGGGCAAACTA	AAATAATGACTGAGGCTCAAAACA	240-292	0.81
D7S2459	CAGAACTATTATTTAGGAG	TAGTAAAACCCATTTGAAG	145-165	0.77
D7S2456	CTGGAAATTGACCTGAAACCTT	ACAGGGGTCTCTCACACATATTA	238-252	0.63
D7S496	AACAACAGTCAACCCACAAT	CTATAACCTCATAANAAACCAAAA	129-141	0.74

## Results

**Fig. 1:**
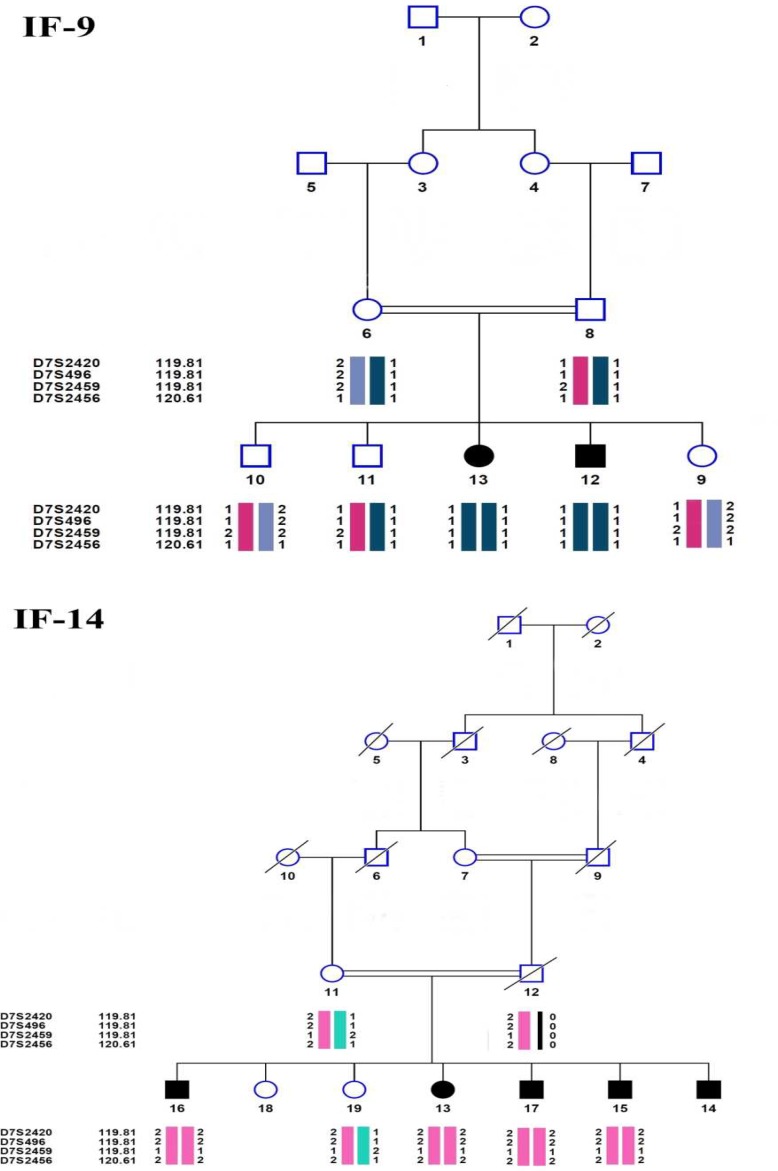
Pedigree and haplotype of Iranian family 9 (IF9) and Iranian family 14 (IF 14). The order of markers is based on the Marshfield map

**Fig. 2:**
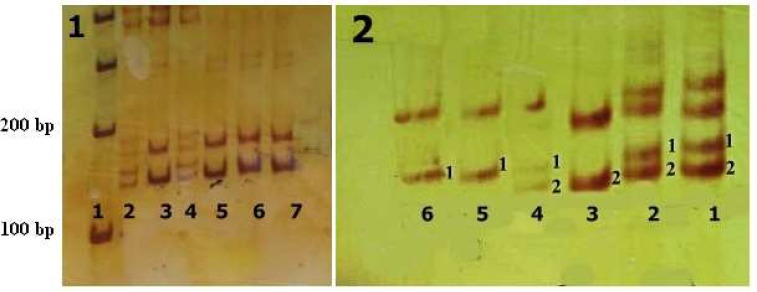
Polyacrylamide gels for D7S2459 marker. Section 1 corresponds to the IF14 (lane 1: size marker, lane 2: mother, lane 4: healthy child, lanes 3, 5, 6 and 7: affected children) and section 2 is related to IF9 (lane1: father, lane 2: mother, lanes 3 and 4: healthy children, lanes 5 and 6: affected children). 200 bp

**Fig. 3:**
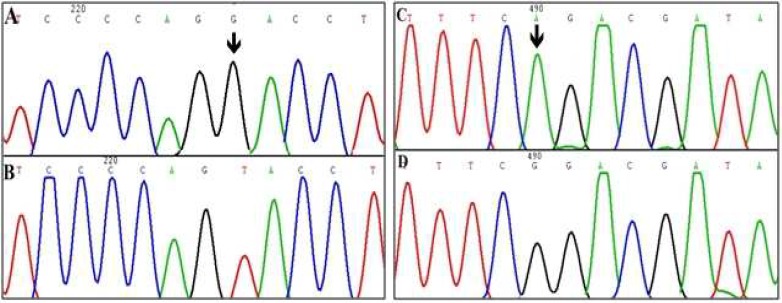
Chromatograms of *SLC26A4* variants. A: normal alleles for c.416 G>T. B: mutant allele of c.416 G>T. C: normal individual for IVS-2 A>G. D: mutant allele for IVS-2 A>G

In this study, most of the subjects displayed bilateral, severe to profound sensorineural HL, and most of families were consanguineous. In haplotype analysis, 2 families from Hamedan province showed linkage to the DFNB4 locus (fig. 1). Two point and multi point LOD and S-link scores related to these families are indicated in Table 2. Linkage to the locus was confirmed by molecular markers on electrophoresis gel (fig. 2). The molecular analysis of the *SLC26A4* gene disclosed 2 variants in exons 7 and 5 in homozygous state in Family 14 and 9, respectively. Exon 5 variant was missense substitution and substitute G for T in 416 location of coding region of *SLC26A4* gene (c.416 G>T) which cause to change glycine to valine (P.G139V). Exon 7 variant is in splicing site which substitute A for G (IVS-2 A>G or c.919-2 A>G) and produce defective protein (fig. 3).

## Discussion

In this investigation, 2 families out of 30 families (~ 7%) were found to be linked to DFNB4 locus (*SLC26A4 *gene). We detected two variants (c.416 G>T and c.919-2 A>G) in two families linked to DFNB4 locus. c.416 G>T is a missense substitution which causes a change of glycine to valine (G139V). This amino acid residue is highly conserved among species and is located in the third transmembrane domain of Pendrin. Thus, structural change can be expected to be deleterious for the protein function. Probably mutant Pendrin proteins are retained in the intracellular region and/ or distributed in endoplasmic reticulum, while wild proteins reach the plasma membrane (20, 21). Sequence analysis of SLC26A4 gene in 46 families in Pakistan showed this mutation as a novel variant (12). The other variant, c.919-2 A>G, was first identified in a Turkish family (22) and subsequently reported in deaf individuals from Japan, Korea and China (13, 23). This mutation causes the removal of splicing site with a resulting stop codon at position 311, resulting in a truncated protein (24).

Other studies concluded that approximately 5% of ARNHL cases in south Asia and other populations are due to mutation in SLC26A4 gene (13-25). Pera et al. in 2007 identified 24 mutations in SLC26A4 gene in 105 Spanish affected subjects and 20 families with ARNHL linked to the locus (26). Lee et al. in 2007 performed DNA sequencing of *SLC26A4* gene in 7 affected subjects with inner ear malformation with EVA and identified 3 different variants. Based on the results of this study, *GJB2* and *SLC26A4* mutations are major causes of congenital hearing loss in Korea (27). In Iran, also several investigations have been carried out to analyze this gene; Sadeghi et al. in 2008 performed linkage analysis of four DFNB loci and mutations in GJB2 in 40 families with ARNHL from west of Iran, 3 families showed linkage to the DFNB4 locus (28). In one study by Kahrizi et al. in 2008, out of 80 Iranian families with ARNHL, 12 were found to be linked to DNBF4 locus, 8 mutations were detected by DNA sequencing of *SLC26A4* gene (9). Tabatabaeifar et al. in 2009 investigated 30 families with non-syndromic HL using linkage analysis and found that 3 families were linked to the DFNB4, Therefore, the frequency of this locus in non-sydromic, HL was estimated to be 10% (16). Recently in a research by Yazdanpanahi et al., out of 30 studied families, 3 (10%) were found to be linked to DFNB4 (19).

**Table 2:** S-Link and LOD scores calculated for families linked to DFNB4

Family	S-LINK	Two point LOD score	Multipoint LOD score
Iranian Family 14 (IF14)	3.28	2.25	2.61
Iranian Family 9 (IF9)	1.45	1.08	1.17

Identified mutations in this study were either missense which alters one amino acid resulting in the loss of proper function of protein or splice mutation which leads to defective protein. Up to date, more than 200 mutations in *SLC26A4* gene have been identified. The majority of these mutations are missense, each of them affecting the different stages of transcription, translation, processing or protein function. Different studies have revealed that mutations associated with PS cause loss of transporting ability of protein while mutations associated with DFNB4 affect the protein function (such as reducing protein function) (29, 30).

According to the studies carried out in different populations worldwide as well as Iran, SLC26A4 ranked as the second cause of ARNHL. Despite extensive studies on DFNB4 locus and *SLC26A4* gene, still there are so many ambiguities about different mutations in this gene and their frequency in Iran that may be due to Iranian different ethnicities, allelic heterogeneousity and the large size of the gene. Therefore, these studies help in further understanding of this gene for clinical decision making and counseling of members of affected families.

Our present investigation illustrates that in 7% of studied families, HL was associated with *SLC26A4* gene. However, the cause of HL in the remaining 93% families is still unknown and more extensive studies may be necessary. Correspond-ingly, in order to gain more information on the exact molecular bases of HL, further studies on the different populations and other loci for the same populations have to be performed.
